# Case of Abcess in the Walls of the Uterus, Communicating with the Rectum

**Published:** 1843-04

**Authors:** Frederic Bird


*Case of Abscess in the Walls of the Uterus, communicating with the Rectum.*—Related at the Westminster Medical Society, December 3, 1842, by Dr. Frederic Bird.—Mrs. G., aged 37, had, previously to the last three years, enjoyed general good health, menstruating regularly. At this date she married, and was soon afterwards attacked with acute deep-seated pain in the hypogastric region, radiating to all parts of the pelvis, and increased by micturition and defecation. These symptoms were associated with general constitutional disturbance, and, in fact, with all the ordinary symptoms of inflammation affecting the uterus. She passed through the usual forms of treatment, and although the more urgent symptoms were mitigated, yet she continued to suffer during the three following months from occasional pain in the region of the uterus, always produced by attempts at expelling the contents of the bladder or rectum, the discharge of fæces being also sometimes effected with great difficulty. An internal examination made at this period detected the uterus lower in the vagina than usual; there existed marked enlargement of that organ, the chief increase in size being found to occupy the posterior wall; the os and cervix uteri were painful to the touch and tumid.

Shortly after the vaginal examination had been made, about half an ounce of pus suddenly escaped from the rectum, and she experienced immediate relief from her former symptoms. She now became the subject of diarrhœa, generally passing from six to eight evacuations daily, each of which contained more or less purulent matter; pain in micturition was no longer felt, but she invariably suffered greatly when passing motions. The diarrhœa could not be arrested by any of the remedies employed; her general health, nevertheless, slowly improved, and she went into the country, where she remained during the succeeding two years, little or no variation in her symptoms having occurred. The diarrhœa, and with it the discharge of pus from the rectum, continued; on some occasions more than a pint of pus has been thus evacuated during twenty-four hours, and she observed that whenever the pus failed to be discharged so freely as usual the local pain became aggravated. During the whole of this period menstruation had been very irregular, generally occurring at intervals of eight or nine days, accompanied by much lumbar pain and the passage of coagula.

After the lapse of the time mentioned, she again applied to Dr. Bird, suffering from nearly all her previous symptoms, and, in addition to them, profuse menorrhagia; the pain in the region of the uterus was extremely acute, increased by the passage of the fæces and by pressure on the lower part of the abdomen, to which became added a neuralgic condition of the genital organs, the slightest pressure upon which produced extreme suffering; so great was the pain thus excited that she was accustomed to employ a mechanical contrivance to prevent the bedclothes from touching the pubes. A vaginal examination was, with much difficulty and pain, again made; the uterus was found to be nearly in the same state as before, excepting that it had become quite immoveable, appearing as if impacted in the pelvis, just as may be observed in some forms of malignant disease affecting that organ. No benefit resulted from medical treatment, occasional relief only being afforded by large doses of opium and the external application of belladonna.

She continued to suffer from frequent discharges of blood from the vagina, and from all her former symptoms, until the lapse of six weeks, when she sank exhausted by the extreme suffering produced by her disease.

*A post-mortem examination* was made twenty-four hours after death. On laying open the abdomen, the omentum, small intestines, and all the pelvic viscera, were found agglutinated together by peritoneal adhesions of old date. On raising the uterus it was seen to be firmly attached by its upper and posterior portion to the rectum; it presented an irregular form, having the fundus enlarged to about thrice its natural size. A longitudinal section showed this enlargement to have been produced by an abscess seated in the substance of the wall of the fundus uteri, the cavity of which contained about an ounce of dark thick pus; the walls of the abscess varied in thickness from one to three-quarters of an inch, the thinnest portion being nearest to the cavity of the uterus. A communication by means of a short sinus could be traced from the cavity of the abscess to the adherent portion of the rectum, and opening into that intestine by an aperture sufficiently large to admit of the passage of a thick probe, and evidently of old formation. No communication existed between the uterine cavity and that of the abscess. The os and cervix presented no evidence of malignant disease. The Fallopian tubes and ovaries were adherent to the uterus, and could with difficulty be distinguished. The uterus had never been impregnated.

He (Dr. B.) had been induced to bring the case before the notice of the society chiefly from the rarity of such forms of disease; very few cases had as yet been described, and those which he had hitherto met with in the works of Madam Boivin and others were complicated with carcinoma or other malignant disease of the uterus. In the instance he had related no evidence of such disease existed, but it was clearly a case of inflammation of the substance of the uterus terminating in the formation of abscess. It was difficult to account for the neuralgic state of the generative organs, unless the immoveable and apparently impacted condition of the uterus might be received as sufficient to produce such an effect by mechanically pressing upon the surrounding nerves, an idea favored by the fact that at the earlier period of the case, when the uterus was not thus fixed, the pain was limited to that viscus and did not extend to the external organs.

*** The preparation was exhibited to the society, where Dr. Chowne stated that he had never seen a similar case. The uterus and its appendages, he said, were in that conglomerated state which is usually observed in women who had led an irregular life, and which was, probably, in them, dependent upon early and excessive excitement. This adhesion of the ovaries, Fallopian tubes, &c., in prostitutes, had been noticed also in France. Perhaps, he added, in Dr. Bird's case, this condition might have been the result of the disease under which the patient labored. On the same occasion another member of the society, Dr. Reid, said that he also was unacquainted with the record of any similar case, excepting those related by Madame Boivin, and thought the disease must be a remarkably rare one.—*Lond. Lan.*, Jan. 28, 1843.

